# Supplementary material for: Rural-Urban Disparities in Vaccine Hesitancy among Adults in South Tyrol, Italy
Source: Vaccines (Basel). 2022 Nov 5;10(11):1870. doi: 10.3390/vaccines10111870 (PMC9692501; doi:10.3390/vaccines10111870)
Supplement: Supplementary file 1 [file vaccines-10-01870-s001.zip › vaccines-2009309-supplementary.pdf]

vaccines

MDPI

Supplementary Material

Rurality and Vaccine Hesitancy in a Probability-Based Cross-Sectional Survey of South Tyrol, Italy

Verena Barbieri, Christian J. Wiedermann, Stefano Lombardo, Barbara Plagg, Timon Gärtner, Dietmar Ausserhofer, Wolfgang Wiedermann, Adolf Engl, and Giuliano Piccoliori

Table S1. Baseline characteristics for differences between urban and rural residents for the groups of hesitant and non-hesitant participants.

Variable		Urban / Rural				Non-hesitant / Hesitant				
		Non-hesitant N = 1,204		Hesitant N=222			Urban N=602		Rural N=824	
		N (%#)	*p*–Value*	N (%#)	*p*–Value*		N (%†)	*p*–Value*	N (%†)	*p*–Value*
**Age (years)**			n.s.		0.001			0.005		<0.001
18 – 34	urban	108 (20.6)		22 (28.9)		non-hesitant	108 (20.6)		140 (20.6)	
	rural	140 (20.6)		63 (43.8)		hesitant	22 (28.9)		63 (43.8)	
35 – 49	urban	118 (22.5)		20 (26.3)		non-hesitant	118 (22.5)		163 (24.0)	
	rural	163 (24.0)		53 (36.8)		hesitant	20 (26.3)		53 (36.8)	
50 – 64	urban	150 (28.6)		27 (35.5)		non-hesitant	150 (28.6)		192 (28.3)	
	rural	192 (28.3)		22 (15.3)		hesitant	27 (35.5)		22 (15.3)	
≥64	urban	148 (28.2)		7 (9.2)		non-hesitant	148 (28.2)		184 (27.1)	
	rural	184 (27.1)		6 (4.2)		hesitant	7 (9.2)		6 (4.2)	
**Gender**			n.s.		n.s.			n.s.		n.s.
Female	urban	272 (51.8)		36 (46.8)		non-hesitant	272 (51.8)		345 (50.8)	
	rural	345 (50.8)		82 (56.6)		hesitant	36 (46.8)		82 56.6)	
Male	urban	253 (48.2)		41 (53.2)		non-hesitant	253 (48.2)		334 (49.2)	
	rural	334 (49.2)		63 (43.4)		hesitant	41 (53.2)		63 (43.4)	
**Education**			<0.001		0.03			n.s.		0.004
Middle school or lower	urban	117 (22.3)		16 (21.1)		non-hesitant	117 (22.3)		161 (23.7)	
	rural	161 (23.7)		22 (15.3)		hesitant	16 (21.1)		22 (15.3)	
Vocational school	urban	103 (19.7)		20 (26.3)		non-hesitant	103 (19.7)		232 (34.1)	
	rural	232 (34.1)		55 (38.2)		hesitant	20 (26.3)		55 (38.2)	
High school	urban	153 (29.2)		24 (31.6)		non-hesitant	153 (29.2)		180 (26.5)	
	rural	180 (26.5)		54 (37.5)		hesitant	24 (31.6)		54 (37.5)	
University	urban	151 (28.8)		16 (21.1)		non-hesitant	151 (28.8)		107 (15.7)	
	rural	107 (15.7)		13 (9.0)		hesitant	16 (21.1)		13 (9.0)	
**Citizenship**			n.s.		n.s.			n.s.		n.s.
Italian	urban	477 (90.9)		67 (88.2)		non-hesitant	477 (90.9)		631 (92.9)	
	rural	631 (92.9)		132 (91.7)		hesitant	67 (88.2)		132 (91.7)	

Other	urban	48 (9.1)		9 (11.8)		non-hesitant	48 (9.1)		48 (7.1)	
	rural	48 (7.1)		12 (8.3)		hesitant	9 (11.8)		12 (8.3)	
**Native Language[1]**			<0.001		<0.001			n.s.		n.s.
German	urban	193 (36.8)		27 (35.1)		non-hesitant	193 (53.1)		547 (80.7)	
	rural	547 (80.7)		112 (77.2)		hesitant	27 (45.5)		112 (77.2)	
Italian	urban	278 (53.1)		35 (45.5)		non-hesitant	278 (36.8)		60 (8.8)	
	rural	60 (8.8)		10 (6.9)		hesitant	35 (35.1)		10 (6.9)	
Ladin	urban	6 (1.1)		1 (1.3)		non-hesitant	6 (1.1)		41 (6.0)	
	rural	41 (6.0)		9 6.29)		hesitant	1 (1.3)		9 (6.9)	
More than one/another language	urban	47 (9.0)		14 (18.2)		non-hesitant	47 (9.0)		30 (4.4)	
	rural	30 (4.4)		14 (9.7)		hesitant	14 (18.2)		14 (9.7)	
**Household / Family structure (more than one answer possible)**										
Single	urban	85 (16.2)	n.s.	14 (18.2)	n.s.	non-hesitant	85 (16.2)	n.s.	121 (17.8)	0.046
	rural	121 (17.8)		16 (11.0)		hesitant	14 (18.2)		16 (11.0)	
Children 0–6 years of age	urban	53 (10.1)	n.s.	9 (11.7)	n.s.	non-hesitant	53 (10.1)	n.s.	84 (12.4)	0.002
	rural	84 (12.4)		32 (22.1)		hesitant	9 (11.7)		32 (22.1)	
Adolescents 7–17 years of age	urban	88 (16.8)	n.s.	15 (19.5)	n.s.	non-hesitant	88 (16.8)	n.s.	135 (19.9)	n.s.
	rural	135 (19.9)		39 (26.9)		hesitant	15 (19.5)		39 (26.9)	
COVID-19 patients at risk[2]	urban	120 (22.9)	n.s.	7 (9.1)	n.s.	non-hesitant	120 (22.9)	0.006	155 (22.8)	0.016
	rural	155 (22.8)		20 (13.8)		hesitant	7 (9.1)		20 (13.8)	
None of the above	urban	195 (37.1)	n.s.	34 (44.2)	n.s.	non-hesitant	195 (37.1)	n.s.	238 (35.1)	n.s.
	rural	238 (35.1)		56 (38.9)		hesitant	34 (44.2)		56 (38.9)	
**Working in the health sector**			n.s.		n.s.			0.046		n.s.
Yes	urban	39 (7.4)		1 (1.3)		non-hesitant	39 (7.4)		37 (5.5)	
	rural	37 (5.5)		7 (4.8)		hesitant	1 (1.3)		7 (4.8)	
No	urban	486 (92.6)		75 (98.7)		non-hesitant	486 (92.6)		641 (94.5)	
	rural	641 (94.5)		138 (95.2)		hesitant	75 (98.7)		138 (95.2)	
**Chronic disease(s)**			n.s.		0.034			0.023		<0.001
Yes	urban	114 (21.8)		8 (10.5)		non-hesitant	114 (21.8)		119 (17.5)	
	rural	119 17.59)		5 (3.4)		hesitant	8 (10.5)		5 (3.4)	
No	urban	410 (78.2)		68 (89.5)		non-hesitant	410 (78.2)		560 (82.5)	
	rural	560 (82.5)		140 (96.6)		hesitant	68 (89.5)		140 (96.6)	

**Relatives or friends died from COVID-19**			n.s.		n.s.			n.s.		n.s.
Yes	urban	57 (10.9)		6 (7.8)		non-hesitant	57 (10.9)		53 (7.8)	
	rural	53 (7.8)		8 (5.5)		hesitant	6 (7.8)		8 (5.5)	
No	urban	467 (89.1)		71 (92.2)		non-hesitant	467 (89.1)		626 (92.2)	
	rural	626 (92.2)		137 (94.5)		hesitant	71 (92.2)		137 (94.5)	
**Economic situation (last 3 months)**			0.02		n.s.			0.013*		<0.001
Better	urban	23 (4.4)		0 (0.0)		non-hesitant	23 (4.4)		20 (2.9)	
	rural	20 (2.9)		0 (0.0)		hesitant	0 (0.0)		0 (0.0)	
The same	urban	385 (73.5)		48 (63.2)		non-hesitant	385 (73.5)		465 (68.5)	
	rural	465 (68.5)		74 (51.4)		hesitant	48 (63.2)		74 (51.4)	
Worse	urban	103 (19.7)		26 (34.2)		non-hesitant	103 (19.7)		182 (26.8)	
	rural	182 (26.8)		63 (43.8)		hesitant	26 (34.2)		63 (43.8)	
Don`t know	urban	13 (2.5)		2 (2.6)		non-hesitant	13 (2.5)		12 (1.8)	
	rural	12 (1.8)		7 (4.9)		hesitant	2 (2.6)		7 (4.9)	

[#] Per variable, all urban and all rural percentages, respectively, result in sum of 100%.
[†] per variable, all hesitant and all non-hesitant percentages, respectively, result in sum of 100%.
* Chi squared test.

Table S2. Attributes of the sample towards compulsory vaccination for non-coronaviruses and comparisons between urban and rural residents.

Variable	Residency	(Rather) No [1-2] N (%)	[3-4] N (%)	(Rather) Yes [5-6] N (%)	*p*-Value Chi-square test
**Do you agree with the decisions taken by the authorities to increase the coverage of compulsory vaccinations?** [1]	Total N=178	221 (15.5)	281 (19.7)	799 (56.1)	n.s.
	Urban N=62	84 (14.0)	118 (19.6)	348 (57.9)	
	Rural N=116	137 (16.6)	163 (19.8)	451 (54.7)	
**How seriously do you think the consequences for your child's health would be if he/she did not have compulsory vaccinations?**	Total	47 (26.4)	70 (39.3)	61 (34.3)	0.033
	Urban	12 (19.4)	21 (33.9)	29 (46.8)	
	Rural	35 (30.2)	49 (42.2)	32 (27.6)	
**Compulsory vaccinations are not necessary because**					
efficacy is not given	Total	124 (69.7)	37 (20.8)	17 (9.6)	n.s
	Urban	46 (74.2)	14 (22.6)	2 (3.2)	
	Rural	78 (67.2)	23 (19.8)	15 (12.9)	
the natural immune system is completely sufficient	Total	117 (65.4)	44 (24.6)	18 (10.1)	n.s.
	Urban	43 (68.3)	16 (25.4)	4 (6.3)	
	Rural	74 (63.8)	28 (24.1)	14 (12.1)	
those diseases no longer exist	Total	139 (78.1)	32 (18.0)	7 (3.9)	n.s.
	Urban	49 (79.0)	10 (16.1)	3 (4.8)	
	Rural	90 (77.6)	22 (19.0)	4 (3.4)	
The whole thing is just a profit for the pharmaceutical industry	Total	126 (70.4)	29 (16.2)	24 (13.4)	n.s.
	Urban	49 (77.8)	9 (14.3)	5 (7.9)	
	Rural	77 (66.4)	20 (17.2)	19 (16.4)	
**Compulsory vaccinations harm, because**					
the risks are greater than the protection	Total	131 (73.6)	31 (17.4)	16 (9.0)	n.s.
	Urban	50 (79.4)	10 (15.9)	3 (4.8)	
	Rural	81 (70.4)	21 (18.3)	13 (11.3)	
vaccines are not sufficiently controlled	Total	125 (69.8)	35 (19.6)	19 (10.6)	n.s.
	Urban	49 (77.8)	11 (17.5)	3 (4.8)	
	Rural	76 (65.5)	24 (20.7)	16 (13.8)	
Some doctors advise against it	Total	126 (70.4)	40 (22.3)	13 (7.3)	n.s.
	Urban	45 (71.4)	14 (22.2)	4 (6.3)	
	Rural	81 (69.8)	26 (22.4)	9 (7.8)	

there have been negative vaccination experiences in my family	Total	146 (82)	22 (12.4)	10 (5.6)	n.s.
	Urban	52 (83.9)	8 (12.9)	2 (3.2)	
	Rural	94 (81)	14 (12.1)	8 (6.9)	
**In the current debate on COVID19, how do you assess the position of compulsory vaccination?**					
My children must receive the protection they need	Total	18 (10.1)	32 (18)	128 (71.9)	n.s.
	Urban	4 (6.3)	8 (12.7)	51 (81)	
	Rural	14 (12.2)	24 (20.9)	77 (67)	
Herd immunity must be guaranteed	Total	16 (8.9)	57 (31.8)	106 (59.2)	n.s.
	Urban	6 (9.5)	15 (23.8)	42 (66.7)	
	Rural	10 (8.6)	42 (36.2)	64 (55.2)	
At the moment, access to compulsory vaccination is difficult	Total	68 (38.2)	77 (43.3)	33 (18.5)	n.s.
	Urban	27 (43.5)	22 (35.5)	13 (21)	
	Rural	41 (35.3)	55 (47.4)	20 (17.2)	
I am concerned about the decline in compulsory vaccination due to the COVID19 pandemic	Total	56 (31.5)	75 (42.1)	47 (26.4)	n.s.
	Urban	18 (29.0)	28 (45.2)	16 (25.8)	
	Rural	38 (32.8)	47 (40.5)	31 (26.7)	
If it were possible to vaccinate your child at your doctor's office, that would be more reassuring	Total	44 (24.7)	61 (34.3)	73 (41)	n.s.
	Urban	11 (17.7)	23 (37.1)	28 (45.2)	
	Rural	33 (28.4)	38 (32.8)	45 (38.8)	
In the current pandemic situation, compulsory vaccinations can wait	Total	106 (59.2)	47 (26.3)	26 (14.5)	n.s.
	Urban	39 (61.9)	15 (23.8)	9 (14.3)	
	Rural	67 (57.8)	32 (27.6)	17 (14.7)	
**Would your child receive compulsory vaccinations? If so, why?** [2]		yes	empty		
I want to protect my child	Total (N=157)	131 (84)	26 (16)		
	Urban (N=58)	51 (88)	7 (12)		
	Rural (N=99)	81 (82)	18 (18)		
It is my responsibility as a parent	Total	106 (67)	51 (33)		
	Urban	38 (66)	20 (34)		
	Rural	68 (69)	31 (31)		
Otherwise, I would receive a sanction	Total	11 (7.1)	146 (92.9)		
	Urban	4 (6.5)	54 (93.5)		
	Rural	8 (8)	92 (92)		

My friends and family expect me to do it	Total	4 (2.2)	154 (97.8)
	Urban	2 (3.5)	56 (96.5)
	Rural	2 (2)	97 (98)
**Would you require your child to have compulsory vaccinations? If not, why?**			
Vaccination involves expenses (loss of income, transport, parking, holidays, ...)	Total (N=21)	1 (5)	20 (95)
	Urban (N=4)	1 (25)	3 (75)
	Rural (N=17)	0 (0)	17 (100)
I don't know where to do it	Total	1 (5)	20 (95)
	Urban	0 (0)	4(100)
	Rural	1 (6)	16 (94)
Others might accuse me of exposing my child to unknown dangers	Total	0 (0)	21 (100)
	Urban	0 (0)	4 (100)
	Rural	0 (0)	17 (100)
I don't trust the authorities with my data	Total	1 (5)	20 (95)
	Urban	0 (0)	4 (100)
	Rural	1 (6)	16 (94)
I don't believe in the existence of those childhood diseases	Total	1 (5)	20 (95)
	Urban	0 (0)	4 (100)
	Rural	1 (6)	16 (94)
I don't believe in the effectiveness of vaccination	Total	9 (43)	12 (57)
	Urban	1 (25)	3 (75)
	Rural	8 (48)	9 (52)
I am afraid that my child will be infected by vaccination or that side effects will occur	Total	16 (75)	5 (25)
	Urban	3 (75)	1 (25)
	Rural	12 (72)	5 (28)
I believe that vaccination is painful	Total	1 (5)	20 (95)
	Urban	1 (25)	3 (75)
	Rural	0 (0)	100 (0)

[1] The option “I don’t’ know” was available and contains the missing percentages.
[2] One could answer the questions with yes or leave them blank. There was no "no", because multiple answers were possible.
Abbreviation: n.s., not significant.

Table S3. Attributes of the sample toward COVID-19 vaccination and comparisons between urban and rural residents.

Variable	Residency	(Rather) Not agree/serious/no [1 – 2] N (%)	[3 – 4] N (%)	(Rather) Agree/serious/no [5 – 6] N (%)	*p*-Value urban vs. rural [1]
**Apart from COVID-19, I think everyone should be vaccinated according to the national vaccine plan**	Total	209 (14.7)	315 (22.1)	901 (63.2)	<0.001
	Urban	70 (11.6)	112 (18.6)	419 (69.7)	
	Rural	139 (16.9)	203 (24.6)	482 (58.5)	
**How seriously do you think the consequences for your daily life would be if you did not get vaccinated for COVID-19?**	Total	404 (28.3)	506 (35.5)	516 (36.2)	0.001
	Urban	143 (23.8)	215 (35.7)	244 (40.5)	
	Rural	261 (31.7)	291 (35.3)	272 (33)	
**Has the COVID-19 pandemic changed your attitude towards compulsory vaccinations?**		**No**	**Yes, I support it more now**	**Yes, I support it less now**	
	Total	1052 (73.8)	279 (19.6)	94 (6.6)	
	Urban	601 (74.5)	118 (19.6)	35 (5.8)	
	Rural	604 (73.3)	161 (19.5)	59 (7.2)	n.s.
**What is your position on the anti-COVID-19 vaccine?**		**(Rather) Not agree/serious/no [1 – 2] N (%)**	**[3 – 4] N (%)**	**(Rather) Agree/serious/no [5 – 6] N (%)**	
I believe the vaccine can help contain the spread of COVID-19	Total	116 (8.1)	188 (13.2)	1122 (78.7)	n.s
	Urban	44 (7.3)	73 (12.1)	485 (80.6)	
	Rural	72 (8.7)	115 (14.0)	637 (77.3)	
If I knew I was already infected with COVID-19, I would not get the vaccine	Total	768 (53.9)	273 (19.2)	383 (26.9)	0.007
	Urban	353 (58.7)	106 (17.6)	142 (23.6)	
	Rural	415 (50.4)	167 (20.3)	241 (29.3)	
If everyone else is vaccinated against COVID-19, then I should not get vaccinated	Total	1091 (76.5)	188 (13.2)	147 (10.3)	n.s.
	Urban	477 (79.2)	69 (11.5)	56 (9.3)	
	Rural	614 (74.5)	119 (14.4)	91 (11)	
If the vaccine was recommended for me, I would do it	Total	171 (12)	160 (11.2)	1093 (76.8)	n.s.
	Urban	61 (10.1)	65 (10.8)	475 (79.0)	
	Rural	110 (13.4)	95 (11.5)	618 (75.1)	
If your doctor recommended a COVID-19 vaccination, how likely would you be to get vaccinated?	Total	183 (12.9)	170 (11.9)	1071 (75.2)	n.s.
	Urban	70 (11.6)	66 (11.0)	465 (77.4)	
	Rural	113 (13.7)	104 (12.6)	606 (73.6)	

**Which of the following statements on COVID19 vaccination do you agree with? COVID-19 vaccination is not necessary because**					
it is not effective	Total	1036 (72.7)	270 (18.9)	120 (8.4)	n.s.
	Urban	453 (75.4)	97 (16.1)	51 (8.5)	
	Rural	583 (70.7)	173 (21.0)	69 (8.4)	
natural herd immunity to virus spread is achieved and is completely sufficient	Total	944 (66.2)	312 (21.9)	170 (11.9)	<0.001
	Urban	431 (71.6)	103 (17.1)	68 (11.3)	
	Rural	513 (62.3)	209 (25.4)	102 (12.4)	
this disease does not exist/is a trivial flu	Total	1150 (80.7)	192 (13.5)	83 (5.)	0.003
	Urban	509 (84.7)	61 (10.1)	31 (5.2)	
	Rural	641 (77.8)	131 (15.9)	52 (6.3)	
the whole thing is just a profit for the pharmaceutical industry	Total	917 (64.4)	323 (22.7)	184 (12.9)	0.003
	Urban	408 (67.9)	136 (22.6)	57 (9.5)	
	Rural	509 (61.8)	187 (22.7)	127 (15.4)	
**Which of the following statements on COVID19 vaccination do you agree with? COVID-19 vaccination is harmful because**					
long-term risks are unknown	Total	472 (33.1)	487 (34.2)	466 (32.7)	n.s.
	Urban	209 (34.8)	204 (33.9)	188 (31.3)	
	Rural	263 (31.9)	283 (34.3)	278 (33.7)	
new vaccines carry additional risks in the RNA	Total	737 (51.7)	529 (37.1)	159 (11.2)	0.006
	Urban	336 (55.9)	213 (35.4)	52 (8.7)	
	Rural	401 (48.7)	316 (38.3)	107 (13)	
Some doctors advise against it	Total	866 (60.8)	384 (26.9)	175 (12.3)	0.037
	Urban	385 (64.1)	141 (23.5)	75 (12.5)	
	Rural	481 (58.4)	243 (29.5)	100 (12.1)	
an obligation to vaccinate certain groups with priority will lead to great socio-political discussions	Total	590 (41.4)	511 (35.9)	324 (22.7)	0.002
	Urban	266 (44.3)	184 (30.6)	151 (25.1)	
	Rural	324 (39.3)	327 (39.7)	173 (21.0)	
**Would you vaccinate for COVID19? If so, why?** [2]		yes	empty		
I want to protect myself	Total (N=1,204)	839 (69.7)	365 (30.3)		n.s.
	Urban (N=525)	358 (68.3)	167 (31.7)		

	Rural (N=679)	480 (70.7)	199 (29.3)	
It is my responsibility as a citizen	Total	923 (76.7)	281 (23.3)	0.001
	Urban	420 (80.1)	105 (19.9)	
	Rural	503 (74.1)	176 (25.9)	
Otherwise, I would receive a sanction	Total	23 (1.9)	1181 (98.1)	n.s.
	Urban	10 (1.8)	515 (98.2)	
	Rural	13 (1.9)	666 (98.1)	
My friends and family expect me to do so	Total	120 (10.0)	1084 (90.0)	n.s.
	Urban	58 (11.0)	467 (89.0)	
	Rural	62 (9.2)	617 (90.8)	
**Would you vaccinate for COVID19? If not, why?** [2]		yes	empty	
Vaccination involves expenses (loss of income, transport, parking, holidays, etc.).	Total (N=222)	8 (3.6)	214 (96.4)	
	Urban (N=77)	1 (1.3)	76 (98.7)	
	Rural (N=145)	7 (4.6)	138 (95.4)	
I do not know where to do it	Total	4 (1.8)	218 (98.2)	
	Urban	4 (5.2)	73 (94.8)	
	Rural	0 (0)	145 (100)	
Others might accuse me of exposing others to unknown dangers	Total	5 (2.4)	217 (97.6)	
	Urban	0 (0.0)	77 (100.0)	
	Rural	5 (3.3)	140 (96.7)	
I don't trust the authorities with my data	Total	28 (12.6)	194 (87.4)	
	Urban	9 (11.0)	68 (68.0)	
	Rural	19 (13.3)	126 (86.7)	
I do not believe in the existence of COVID-19	Total	12 (5.4)	210 (94.6)	
	Urban	6 (7.8)	71 (92.2)	
	Rural	5 (3.7)	140 (96.3)	
I don't believe in the effectiveness of vaccination	Total	113 (50.9)	109 (49.1)	
	Urban	44 (56.8)	33 (43.2)	
	Rural	69 (47.8)	76 (52.2)	
I am afraid of being infected by vaccination or of side effects occurring	Total	146 (65.7)	76 (34.3)	
	Urban	49 (63.6)	28 (35.7)	
	Rural	96 (66.4)	49 (33.6)	

	Total	14 (6.3)	208 (93.7)
I believe that vaccination is painful	Urban	3 (3.4)	74 (96.6)
	Rural	11 (7.3)	134 (92.7)

[1] Urban versus rural, chi-square test.

[2] Reasons for vaccine non-hesitancy and hesitancy. One could answer the questions with yes or leave them blank. There was no "no", because multiple answers were possible.

Abbreviation: n.s., not significant.

Table S4. Attitudes towards COVID-19 disease, vaccine, and vaccination of survey participants by residence and vaccination hesitancy.

Category	Question	Vaccination	Residence			p–Value [1]	
			Total N (%)	Urban N (%)	Rural N (%)	Residence [2]	Hesitancy [3,4]
Decision making	I think the decisions about COVID-19 made by the public authorities are right	COVID-19 (N=1,426)	789 (57.8)	353 (60.4)	436 (55.9)	n.s.	<0.001
		General (N=178)	89 (52.7)	36 (60)	53 (48.6)	n.s.	0.006
	I think the decisions about vaccination against COVID-19 made by the authorities are right	COVID-19	992 (75)	420 (75.5)	572 (74.6)	n.s.	<0.001
		General	118 (72.4)	46 (82.1)	72 (67.3)	0.044	<0.001
	I think the decisions about compulsory general vaccination by the public authorities are right	COVID-19	953 (73.2)	412 (74.9)	541 (71.9)	n.s.	<0.001
		General	110 (67.5)	42 (73.7)	68 (64.2)	n.s.	<0.001
Trust in COVID-19 vaccination [5]	I believe the vaccination can contain the virus	COVID-19	1227 (86.1)	529 (88)	698 (84.7)	n.s.	<0.001
		General	147 (82.1)	53 (84.1)	94 (81)	n.s.	<0.001
	If I knew that I had already been infected with the virus, I wouldn't get vaccinated	COVID-19	492 (34.5)	186 (30.9)	306 (37.1)	0.014	<0.001
		General	75 (42.1)	22 (35.5)	53 (45.7)	n.s.	0.004
	When all the others are vaccinated against the virus, I don't need to get vaccinated	COVID-19	219 (15.4)	81 (13.5)	138 (16.7)	n.s.	<0.001
		General	25 (14.0)	4 (6.3)	21 (18.1)	0.03	0.005
	If vaccination would be recommended for me, I would get vaccinated	COVID-19	1175 (82.5)	509 (84.6)	666 (80.9)	n.s.	<0.001
		General	136 (76.4)	50 (80.6)	86 (74.1)	n.s.	<0.001
	If my doctor recommended a COVID-19 vaccination, I would get vaccinated	COVID-19	1152 (81.0)	497 (82.8)	655 (79.6)	n.s.	<0.001
		General	129 (72.1)	47 (74.6)	82 (70.7)	n.s.	<0.001
COVID-19 vaccination is not necessary, because…	…it is not effective	COVID-19	198 (13.9)	78 (13.0)	120 (14.6)	n.s.	<0.001
		General	30 (16.9)	7 (11.3)	23 (19.8)	n.s.	<0.001
	…natural herd immunity is achieved with virus spread and that is quite sufficient	COVID-19	274 (19.2)	110 (18.3)	164 (19.9)	n.s.	<0.001
		General	42 (23.6)	9 (14.5)	33 (28.4)	0.037	<0.001
	…this disease does not exist/ is a normal flu	COVID-19	149 (10.5)	48 (8.0)	101 (12.3)	0.009	<0.001
		General	25 (14.0)	7 (11.1)	18 (15.5)	n.s.	<0.001
	…the whole thing is only a profit for the pharmaceutical industry	COVID-19	320 (22.5)	117 (19.4)	203 (24.7)	0.019	<0.001
		General	52 (29.2)	15 (24.2)	37 (31.9)	n.s.	<0.001
COVID-19 vaccination is	...long-term risks are not known	COVID-19	665 (46.7)	275 (45.7)	390 (47.4)	n.s.	<0.001
		General	94 (52.8)	31 (50)	63 (54.3)	n.s.	0.001

harmful, because…	…new vaccines pose additional risks to the RNA	COVID-19	306 (21.5)	108 (18.0)	198 (24.0)	0.006	<0.001
		General	48 (27.0)	10 (16.1)	38 (32.8)	0.017	<0.001
	...some doctors advise against it	COVID-19	307 (21.5)	126 (20.9)	181 (22.0)	n.s.	<0.001
		General	49 (27.4)	15 (24.2)	34 (29.1)	n.s.	<0.001
	…a compulsory corona vaccination with prioritization of certain groups will lead to major socio-political discussions	COVID-19	513 (36.0)	218 (36.2)	295 (35.8)	n.s.	<0.001
		General	74 (41.6)	24 (38.7)	50 (43.1)	n.s.	0.003

[1] *p*-values refer to the Chi-square test, in the urban areas a total of N=602 participants answered the questions (525 were non-hesitant and 77 were hesitant), in the rural areas a total of N=824 participants answered the questions (679 were non-hesitant and 145 were hesitant)
[2] Chi-square test null-hypothesis, the question is not answered differently by urban/rural residents.
[3] Vaccine hesitancy was measured with a dichotomous question: "Would you get vaccinated against COVID-19?"
[4] Chi-square test null-hypothesis, the question is not answered differently regarding vaccine hesitancy.
[5] (Rather) agreement, [4 - 6] on Likert scale.
Abbreviations: n.s., not significant

Table S5. Variables related to vaccination hesitancy and the effects of rural versus urban residency in South Tyrol, Italy.

Category	Question	Vaccination	Residence			p–Value [1]	
			Total N 1426/178 [2] N (%)	Urban N 602/62 N (%)	Rural N 824/116 N (%)	Residence [3]	Hesitancy [4,5]
Frequency of search for information on COVID-19? [6]	How often do you search for information on the new Coronavirus?	COVID-19	628 (44.0)[1]	320 (53.2)[1]	308 (37.4)[1]	<0.001	<0.001
		General	56 (31.5)	27 (34.4)	29 (24.7)	n.s.	n.s
General national vaccination schedule	Aside from COVID-19, I think everyone should be vaccinated according to the national vaccination schedule	COVID-19	1045 (73.3)	470 (7.81)	575 (69.8)	<0.001	<0.001
		General	130 (73.0)	80 (69.0)	50 (80.6)	n.s.	<0.001
Trust – How much do you trust information about COVID-19 and vaccinations from the following sources? [7]	TV	COVID-19[2]	475 (33.3)	204 (33.9)	271 (32.9)	n.s.	<0.001
		General[2]	51 (28.3)	19 (30.2)	32 (27.4)	n.s.	n.s
	Newspapers/press	COVID-19	468 (32.8)	204 (33.9)	264 (32.0)	n.s.	<0.001
		General	55 (30.7)	24 (38.1)	31 (26.7)	n.s.	n.s.
	Health care workers	COVID-19	1009 (70.8)	438 (72.8)	571 (69.3)	n.s.	<0.001
		General	129 (72.5)	53 (85.5)	76 (65.5)	0.012	0.025
	Social media	COVID-19	164 (11.5)	79 (13.1)	85 (10.3)	n.s.	0.013
		General	17 (9.7)	11 (17.7)	6 (5.3)	0.022	n.s.
	Radio	COVID-19	534 (37.5)	224 (37.3)	310 (37.6)	n.s.	<0.001
		General	69 (38.8)	29 (46.8)	40 (34.5)	n.s.	0.042
	Ministry of Health	COVID-19	836 (58.7)	397 (65.9)	439 (53.3)	<0.001	<0.001
		General	104 (58.8)	43 (70.5)	61 (52.6)	0.036	0.001
	National Institute of Health	COVID-19	731 (51.3)	378 (62.8)	353 (42.8)	<0.001	<0.001
		General	95 (52.8)	44 (68.8)	51 (44.0)	0.005	0.012
	Famous people/influencers	COVID-19	145 (10.2)	54 (9.0)	91 (11)	n.s.	<0.001
		General	17 (9.5)	5 (7.9)	12 (10.3)	n.s.	n.s.
	WHO	COVID-19	822 (57.7)	368 (61.3)	454 (55.1)	n.s.	<0.001
		General	98 (55.1)	42 (67.7)	56 (48.3)	0.043	<0.001
	Regional toll-free and emergency numbers	COVID-19	616 (43.2)	311 (51.7)	305 (37.0)	<0.001	<0.001
		General	77 (43.0)	36 (57.1)	41 (35.3)	0.019	0.004

	Civil protection	COVID-19	939 (65.9)	417 (69.4)	522 (63.3)	0.007	<0.001
		General	112 (63.3)	46 (74.2))	66 (57.4)	n.s.	0.014
	Provincial government	COVID-19	741 (52.0)	339 (56.4)	402 (48.8)	0.012	<0.001
		General	89 (49.4)	40 (63.5)	49 (41.9)	0.016	n.s.
	Management of the South Tyrolean Health Service	COVID-19	550 (38.6)	185 (30.8)	365 (44.3)	<0.001	<0.001
		General	70 (39.3)	16 (25.8)	54 (46.6)	0.005	0.001
Conspiracy Thinking [8]	I think that many very important things happen in the world, which the public is never informed about	COVID-19	952 (66.8)	383 (63.6)	569 (69.1)	0.030	<0.001
		General	124 (70.1)	42 (67.7)	82 (71.3)	n.s.	n.s.
	I think politicians usually do not tell us the true motives for their decisions	COVID-19	803 (56.4)	340 (56.6)	463 (56.2)	n.s.	<0.001
		General	100 (55.9)	35 (55.6)	65 (56.0)	n.s.	<0.001
	I think that government agencies closely monitor all citizens	COVID-19	407 (28.6)	167 (27.8)	240 (29.1)	n.s.	<0.001
		General	61 (34.3)	20 (32.3)	41 (35.3)	n.s.	0.019
	I think events that superficially seem to lack a connection superficially are often the result of secret activities	COVID-19	424 (29.8)	153 (25.5)	271 (32.9)	0.002	<0.001
		General	54 (30.3)	19 (30.6)	35 (30.2)	n.s.	0.004
	I think that there are secret organizations that greatly influence political decisions	COVID-19	509 (35.7)	203 (33.8)	306 (37.1)	n.s.	<0.001
		General	64 (36.0)	21 (33.9)	43 (37.1)	n.s.	0.031
Resilience	I have a hard time making it through stressful events	COVID-19	445 (31.2)	163 (27.1)	282 (34.2)	0.004	n.s.
		General	54 (30.3)	32.3 (62)	34 (29.3)	n.s.	n.s.
	It does not take me long to recover from a stressful event	COVID-19	751 (52.7)	306 (50.8)	445 (54.0)	n.s.	n.s.
		General	97 (54.2)	30 (47.6)	67 (57.8)	n.s.	n.s.
	It is hard for me to snap back when something bad happens	COVID-19	497 (34.9)	198 (32.9)	299 (36.3)	n.s.	n.s.
		General	64 (36.0)	21 (33.9)	43 (37.1)	n.s.	n.s.
Altruism [9]	I enjoy doing things for others	COVID-19	1145 (80.4)	480 (79.9)	824 (80.7)	n.s.	n.s.
		General	143 (80.3)	48 (77.4)	95 (81.9)	n.s.	n.s.
	I try to help others, even if they do not help me	COVID-19	1085 (76.1)	457 (75.9)	628 (76.3)	n.s.	0.004
		General	131 (73.6)	43 (69.4)	88 (75.9)	n.s.	n.s.
	Seeing others prosper makes me happy	COVID-19	1221 (85.6)	525 (87.2)	696 (84.5)	n.s.	n.s.
		General	154 (86.5)	54 (87.1)	100 (86.2)	n.s.	n.s.
	I care about the needs of other people	COVID-19	1069 (75.0)	455 (75.7)	614 (74.5)	n.s.	n.s.
		General	140 (78.2)	50 (79.4)	90 (77.6)	n.s.	n.s.
		COVID-19	222 (15.6)	79 (13.1)	143 (17.4)	0.03	n.s.

	I come first and should not have to care so much for others	General	28 (15.7)	10 (16.1)	18 (15.5)	n.s.	n.s.
Well-being	**In the last two weeks I was…**						
	…happy and in a good mood	COVID-19	490 (34.4)	225 (37.4)	265 (32.2)	0.041	0.005
		General	59 (33.1)	23 (37.1)	36 (31.0)	n.s.	0.017
	…calm and relaxed	COVID-19	489 (34.3)	235 (39.1)	254 (30.8)	0.001	n.s.
		General	58 (32.6)	23 (37.1)	35 (30.2)	n.s.	n.s.
	…active and energetic	COVID-19	589 (41.3)	280 (46.5)	309 (37.5)	0.001	n.s.
		General	73 (41.2)	31 (50)	73 (63.5)	n.s.	n.s.
	…I woke up fresh and rested	COVID-19	559 (39.2)	267 (44.4)	292 (35.4)	0.001	0.001
		General	84 (47.2)	35 (56.5)	49 (42.2)	n.s.	n.s.
	…my everyday life was full of things that interest me	COVID-19	628 (44.1)	287 (47.7)	341 (41.4)	0.019	n.s.
		General	79 (44.4)	27 (43.5)	52 (44.8)	n.s.	n.s.

[1] *p*-values refer to the Chi-square test, in the urban areas a total of N=602 participants answered the questions (525 were non-hesitant and 77 were hesitant), in the rural areas a total of N=824 participants answered the questions (679 were non-hesitant and 145 were hesitant).
[2] N, number of all participants/number of participants with children <6 years of age.
[3] Chi-square test for null hypothesis 'The question is not answered differently by participants of urban/rural residency'.
[4] Vaccine hesitancy was measured with a dichotomous question: "Would you get vaccinated against COVID-19?"
[5] Chi-square test for null hypothesis 'The question is not answered differently by hesitant and non-hesitant'.
[6] More than once a week (Likert scale 1 – 3).
[7] 'Don't know' answers were available for items regarding trust in information sources.
[8] Conspiracy Mental Questionnaire [26].
[9] Altruistic Attitudes Among Older Adults Scale [28].
Abbreviations: n.s., not significant; n.a., not applicable.
